# Current Concepts of Mechanisms in Drug-Induced Hepatotoxicity

**DOI:** 10.2174/092986709788803097

**Published:** 2009-08

**Authors:** Stefan Russmann, Gerd A Kullak-Ublick, Ignazio Grattagliano

**Affiliations:** 1 Division of Clinical Pharmacology and Toxicology, University Hospital Zurich, Zurich, Switzerland; 2 General and Internal Medicine, Department of Internal and Public Medicine, University of Bari, Bari, Italy

**Keywords:** Acetaminophen, apoptosis, death receptors, drug-induced liver injury, drug metabolism, Fas, glutathione, hepatotoxicity, metabonomics, mitochondria, mitochondrial permeability transition, necrosis, TNF alpha.

## Abstract

Drug-induced liver injury (DILI) has become a leading cause of severe liver disease in Western countries and therefore poses a major clinical and regulatory challenge. Whereas previously drug-specific pathways leading to initial injury of liver cells were the main focus of mechanistic research and classifications, current concepts see these as initial upstream events and appreciate that subsequent common downstream pathways and their attenuation by drugs and other environmental and genetic factors also have a profound impact on the risk of an individual patient to develop overt liver disease. This review summarizes current mechanistic concepts of DILI in a 3-step model that limits its principle mechanisms to three main ways of initial injury, i.e. direct cell stress, direct mitochondrial impairment, and specific immune reactions. Subsequently, initial injury initiates further downstream events, i.e. direct and death receptor-mediated pathways leading to mitochondrial permeability transition, which then results in apoptotic or necrotic cell death. For all mechanisms, mitochondria play a central role in events leading to apoptotic vs. necrotic cell death. New treatment targets consequently focus on interference with downstream pathways that mediate injury and therefore determine the ultimate outcome of DILI. Genome wide and targeted pharmacogenetic as well as metabonomic approaches are now used in order to reach the key goals of a better understanding of mechanisms in hepatotoxicity, and to develop new strategies for its prediction and treatment. However, the complexity of interactions between genetic and environmental risk factors is considerable, and DILI therefore currently remains unpredictable for most hepatotoxins.

## INTRODUCTION

The liver may be considered as the most important organ in drug toxicity for two reasons: on the one hand it is functionally interposed between the site of absorption and the systemic circulation and is a major site of metabolism and elimination of foreign substances; but on the other hand these features also render it a preferred target for drug toxicity. Drug-induced liver injury (DILI) therefore poses a major clinical problem. DILI has become the leading cause of acute liver failure and transplantation in Western countries [1, 2]. Intrinsic hepatotoxicity following acetaminophen (APAP; paracetamol in Europe) overdose accounts for the majority of cases of drug-induced acute liver failure in the United States and the United Kingdom. In contrast, hepatotoxicity associated with most other drugs is idiosyncratic, which implies by definition that DILI develops in only a small proportion of subjects exposed to a drug in therapeutic doses, and the risk of acute liver failure associated with idiosyncratic hepatotoxins is usually less than 1 per 10’000 exposed patients. However, more than 1’000 drugs and herbal products have been associated with idiosyncratic hepatotoxicity [3-6], and taken together idiosyncratic hepatotoxicity is responsible for more than 10% of all cases of acute liver failure [1, 2]. DILI also represents a major challenge for industry and regulatory authorities: it is a leading cause for termination of further substance development in preclinical and clinical phases, and it is also the most common single adverse drug reaction leading to refusal of market approval. However, in many instances a drug’s hepatotoxic potential can only be recognized postmarketing, and DILI has therefore also been the most frequent single reason for withdrawing drugs from the market and frequently requires modification of labeling [7, 8].

One of its most intriguing features is the fact that DILI can mimic all forms of acute or chronic liver disease. Another aspect of particular interest is the unpredictable idiosyncratic occurrence of DILI and the chemical heterogeneity of hepatotoxins causing idiosyncratic DILI. These aspects already indicate the wide range of targeted structures and cell types, the diversity of the involved mechanisms, and the importance of individual risk factors. During recent years the increasing insight into the complex processes and sequence of events involved in liver cell injury in general and DILI in particular had a substantial impact on current approaches to mechanistic hepatotoxicity and the subsequent definition of future research targets. Whereas previously drug-specific pathways leading to the initial injury of liver cells were the main focus of mechanistic research and classifications, current concepts see these as so-called initial “upstream” events and appreciate that subsequent common “downstream” pathways and their activation or inhibition by drugs and other environmental and genetic factors also have a profound impact on the risk of an individual patient to develop overt liver disease. In particular, these concepts emphasize the central role of mitochondria, of events leading to apoptotic vs. necrotic cell death, and of factors that balance injurious vs. protective and regenerative responses towards initial cell stress.

This review will therefore focus on these new concepts and their implication for our understanding of mechanisms and risk factors including their complex interactions, as well as for the development of new treatments in DILI.

## GENERAL MECHANISMS OF HEPATOTOXICITY

DILI is commonly classified into intrinsic vs. idiosyncratic hepatotoxicity, and the latter further into allergic vs. non-allergic. Intrinsic hepatotoxicity is regarded as dose-dependent and predictable above an approximate threshold dose, whereas idiosyncratic hepatotoxicity occurs without obvious dose-dependency and in an unpredictable fashion. Allergic idiosyncratic hepatotoxicity is characterized by the presence of typical symptoms and signs of adaptive immune reactions, including fever, skin reactions, eosinophilia, formation of autoantibodies, and a short latency time particularly after re-exposure. Other clinical classifications differentiate e.g. between hepatocellular, cholestatic or mixed liver enzyme patterns, histological criteria, acute vs. chronic onset, or severity. These classifications are useful in clinical practice because they describe typical clinical signatures of DILI for specific drugs, and they can also give useful hints regarding the involved mechanisms. Nevertheless, one must realize that these classifications are descriptive and based on clinical or histopathological criteria. They can therefore be misleading if mixed with mechanistic concepts and may indeed be the foundation of some classic paradigms that are now challenged by recent advances in mechanistic hepatotoxicity. For example, common misconceptions are that specific substances can be clearly classified as either intrinsic *or* idiosyncratic hepatotoxins, and that dose or direct cell injury play no role in idiosyncratic hepatotoxicity. However, unpredictable rare severe DILI often develops on a background of frequent and dose-dependent mild increases in transaminases. Isoniazid is an example for such a hepatotoxin that is associated with mild intrinsic as well as severe idiosyncratic occurrence of DILI. On the other hand APAP, considered as a classic intrinsic hepatotoxin in doses above 10 to 15 g per day, may cause severe liver disease in therapeutic doses below 4 g per day in some patients [9, 10], whereas other patients tolerate very high doses without problems [11]. And even if no clear dose-response relationship can be established for drugs associated with idiosyncratic DILI, idiosyncratic hepatotoxicity is extremely rare for any drug in doses below 10 mg per day [12, 13], and allergic halothane-induced hepatotoxicity occurs more frequently at higher doses [14].

A major challenge for mechanistic classifications is that DILI is not sufficiently characterized by the initial injury, but always involves several mechanisms, regulatory systems and risk factors with complex interactions; this also explains why for most hepatotoxins there are no experimental models available, a drug’s hepatotoxic potential is often not recognized before marketing, the exact contribution of different processes leading to DILI in humans remain largely unknown, and targeted treatments of DILI are not available except for APAP-induced hepatotoxicity. A possible approach to this dilemma is a general working model that integrates the principle initial specific mechanisms of toxic liver cell injury with recently gained knowledge of the unspecific complex regulation of injurious vs. protective processes involved in liver cell injury.

## A GENERAL 3-STEP MODEL FOR DRUG-INDUCED LIVER INJURY

Such a general mechanistic model is presented in Fig. (**1**). According to this model DILI involves three subsequent main steps. It also includes details on the intrinsic and extrinsic pathways emphasizing the central role of mitochondria for the mechanisms leading to apoptosis vs. necrosis. Fig. (**2**) additionally demonstrates the important role of risk factors in DILI.

### 1. Initial Mechanisms of Toxicity: Direct Cell Stress, Direct Mitochondrial Impairment, and Specific Immune Reactions

First, drug metabolites or less frequently also parent drugs cause direct cell stress, target mitochondrial function, or trigger specific immune reactions. The most important drug metabolizing enzyme system for the creation of hepatotoxic reactive metabolites is the polymorphic cytochrome P450 (CYP450) family that mediates oxidative phase-I drug metabolism. However, conjugative phase-II metabolism may also result in hepatotoxic metabolites, e.g. acyl glucuronides are well known to cause DILI [15, 16].

Reactive metabolites can exert initial cell stress through a wide range of mechanisms including depletion of glutathione (GSH), or binding to enzymes, lipids, nucleic acids and other cell structures. Furthermore reactive metabolites or parent drugs may specifically inhibit other hepatocellular functions such as the apical (canalicular) bile salt efflux pump (BSEP, *ABCB11* gene), in which case the subsequent intracellular accumulation of its substrates may cause secondary toxic hepatocyte damage [17].

In case of initial targeting of mitochondria, reactive metabolites or parent drugs uncouple or inhibit the mitochondrial respiratory chain causing ATP depletion and increased concentrations of reactive oxygen species (ROS), inhibit β-oxidation leading to steatosis (e.g. after intramitochondrial accumulation of amiodarone), damage mitochondrial DNA or interfere with its replication, or directly cause mitochondrial permeability transition (MPT), i.e. opening of the “MPT pore” located in their inner membrane [18-21]. There is probably an injury threshold that involves inhibition of mitochondrial electron transport below a critical threshold and an increase in cytosolic ROS and JNK activation above a critical threshold for liver injury to take place. The inhibition of mitochondrial electron transport in the early stages will not be reflected by elevated ALT values, indicating the requirement for earlier markers of impending mitochondrial damage. Functional tests such as the ^13^C-methionine breath test or the use of NMR spectra for metabonomics may prove to be useful here [22-24].

Specific immune responses involving cytotoxic T-cells with concomitant release of inflammatory cytokines can be evoked by reactive metabolites that covalently bind to proteins and are subsequently recognized as neo-antigens (hapten formation). Their subsequent major histocompatibility complex (MHC)-dependent presentation on antigen presenting cells may then activate formation of antibodies against haptens or autoantibodies against cell structures such as CYP450 enzymes [25, 26].

In some instances initial injury also targets nonparenchymal liver cells. Examples include toxicity against biliary epithelial cells by reactive flucloxacillin metabolites [27], direct activation of stellate cells by methotrexate leading to fibrosis [28], or sinusoidal toxicity by herbal pyrrolizidine alkaloids or chemotherapy used for hematopoietic stem cell transplantation causing sinusoidal obstruction syndrome (venoocclusive disease) [29]. Further discussion of such nonparenchymal hepatotoxicity is beyond the scope of this review, but if the resulting damage is sufficiently severe it may eventually also extend to hepatocytes and lead to acute or chronic liver failure.

Different hepatotoxins are typically associated with specific patterns regarding their initial mechanism of injury. However, one should be aware of the fact that a single drug may concomitantly act through several of these initial mechanisms, and that for many drugs at least some of the involved mechanisms currently remain unknown.

These initial specific injurious mechanisms can also be referred to as “upstream events”. In the next step they lead to subsequent rather unspecific “downstream events” that involve the innate immune system, which balances pro- and anti-inflammatory responses and therefore determines the further progress to severe injury or recovery.

### 2. Direct and Death Receptor-Mediated Pathways Leading to Mitochondrial Permeability Transition

Second, initial cell stress and/or initial specific immune reactions lead to MPT. If the initial mechanism does not directly target and impair mitochondrial function, this occurs in two principle ways, i.e. either *via* a direct pathway initiated by severe cell stress (intrinsic pathway), or *via* an indirect death receptor-amplified pathway that is triggered by mild cell stress and/or specific immune reactions (extrinsic pathway) [30].

In the intrinsic pathway severe intracellular stress activates the endoplasmic reticulum pathway, lysosomal permeabilization, or c-jun N-terminal kinase (JNK) [31-34]. Subsequent activation of pro-apoptotic (e.g. Bax, Bak, Bad) and inhibition of anti-apoptotic (e.g. Bcl-2, Bcl-xL) proteins of the Bcl-2 family then activates MPT.

In the extrinsic pathway an initial mild injury may be amplified if inflammatory responses due to mild stress and/or additional factors have modulated the innate immune system, where signaling cytokines that promote (e.g. IL-12) or prevent (e.g. IL-4, IL-10, IL-13, MCP-1) injury are usually well balanced. As a consequence, sensitized liver cells become more susceptible to lethal effects of tumor necrosis factor alpha (TNFα), Fas ligand (FasL) and interferon gamma (IFγ). This is particularly important if one considers that the liver as the central organ of detoxification is constantly exposed to cell stress that can activate TNFα and FasL. If the initial event is a specific immune reaction, MHC-dependent antigen presentation will activate the release of TNFα and FasL from Kupffer cells (hepatic macrophages) and cytotoxic T-cells. According to the danger hypothesis for autoimmune diseases, haptenization alone may be insufficient to trigger the development of frank allergic hepatotoxicity, which requires an additional stimulation, a so-called danger-signal [12]. If reactive metabolites cause concomitant mild direct cell stress or if other concomitant inflammatory diseases are present, the accompanying release of injurious cytokines may constitute such a danger signal that promotes MHCII-dependent antigen presentation, renders hepatocytes more susceptible to injury, and therefore promotes autoimmune hepatotoxicity. Regardless of how the extrinsic pathway is initiated, eventually TNFα and FasL bind to intracellular death receptors, and TNF and Fas receptor-associated death domain proteins (TRADD/FADD) will subsequently activate initiator caspase 8. The activating death-receptor complex is also called the death-inducing signaling complex (DISC). Although initiator caspase 8 can start apoptosis through a direct activation of effector caspases 3, 6 and 7, this direct path appears to be too weak in hepatocytes to mediate apoptosis [35]. Therefore an amplification mechanism is required: caspase 8 can activate pro-apoptotic Bcl-2 proteins (e.g. Bid), as well as signaling ceramides. Like in the intrinsic pathway this leads to MPT, which therefore plays a key role and is a common step that mediates further cell death in both, intrinsic as well as extrinsic pathways [36].

### 3. Apoptosis and Necrosis

Third, impaired mitochondrial function and energy production leads to apoptotic or necrotic cell death. MPT allows massive influx of protons through the inner mitochondrial membrane, which stops mitochondrial ATP synthesis. Mitochondrial ATP depletion resulting from MPT (or other direct mechanisms of mitochondrial damage mentioned above) causes matrix expansion and mitochondrial outer membrane permeabilization and rupture with release of cytochrome c and other pro-apoptotic mitochondrial proteins from the intermembrane space into the cytosol [30, 37-39].

In the case of apoptosis, cytochrome C then binds to a cytoplasmic scaffold (apaf-1) and pro-caspase 9, forming a complex called apoptosome, which activates signaling procaspase 9. This, however, is an active process that requires ATP and can therefore only start if MPT did not rapidly and simultaneously occur in all mitochondria. Only if some mitochondria are left intact and continue to synthesize ATP, activated pro-caspase 9 and possibly also other pro-apoptotic mitochondrial proteins subsequently activate executioner caspase 3. Caspase 3 will then cleave specific cell proteins and further activate pro-caspases 6,7 and 2, which have their own target proteins [40]. These processes eventually result in programmed apoptotic cell death, which is characterized by cytoplasmic and nuclear condensation and fragmentation without loss of membrane integrity. Histologically these remaining fragments are referred to as Councilman bodies. Apoptosis equals a “silent” cell death, where apoptotic fragments are removed by phagocytosis with little accompanying inflammation and therefore also only little secondary damage.

Necrosis, in contrast, develops if the initial injury is so severe that MPT quickly occurs in all mitochondria, or if other mechanisms cause rapid severe mitochondrial ATP depletion, preventing the apoptotic pathway. This is typical for hepatotoxins that directly cause profound initial cell stress. However, in the absence of ATP also activation of the extrinsic pathway may lead to necrotic cell death. Cell swelling and lysis that follow severe disturbance of cell functions characterize necrosis. Necrotic cell lysis induces inflammatory responses including release of cytokines, which is important because these may amplify initial injury through a sensitization of surrounding hepatocytes and therefore cause further collateral damage.

Finally it should be mentioned that the distinction between apoptosis and necrosis is not always clear-cut. Mixed phenomena have been described, and the same hepatotoxin may cause one or the other, or even the concomitant occurrence of both, depending on the circumstances including dose and preexisting vulnerability of hepatocytes [41, 42]. Apoptosis and necrosis may therefore also be regarded as a continuous spectrum. Furthermore some controversy exists around the exact mechanisms and triggers for hepatotoxic apoptosis including the role of MPT.

In conclusion one can state that mitochondria stand in the center of life and death in hepatotoxicity: they can be targets of initial direct toxicity, MPT plays a key role in the further signaling of extrinsic and intrinsic pathways, and because mitochondria generate most of the cell’s ATP supply and are also the main intracellular source of oxygen and nitrogen free radicals, the extent of mitochondrial impairment finally determines whether hepatocytes die by apoptosis or necrosis [43].

#### Protective Mechanisms and Regeneration

Additional factors and pathways modulate intrinsic and extrinsic pathways and the subsequent induction of apoptosis and necrosis. In addition to its DISC-mediated injurious effects, TNFα on the other hand also induces nuclear transcription factor kappa B (NF-κB)-dependent survival gene expression. NF-κB responsive genes inhibit JNK and promote upregulation of antioxidant genes [44]. Similarly, in response to oxidative stress nuclear factor erythroid-derived 2-like (NFE2L, also called Nrf2) upregulates antioxidant genes that protect against cell stress [45]. Particularly, the antioxidant glutathione (GSH) plays a key role for cell protection against DILI. GSH is an important scavenger of ROS of various origins in mitochondria and cytosol, but also of reactive metabolites, e.g. N-acetyl-p-benzoquinone imine (NAPQI), the toxic metabolite of APAP formed by CYP450 2E1. Accordingly, replenishment of GSH stores is the principle protective mechanism for treatment of APAP-intoxication with the GSH precursor N-acetylcysteine. Furthermore GSH appears to indirectly modify cell injury through an effect on other injurious or protective mechanisms, such as the requirement of high GSH levels for NF-κB-activated survival gene expression [46, 47], or protection against Fas-induced apoptosis [48]. As mentioned above, also the innate immune system, particularly natural killer T-cells, can have both, injurious as well as protective functions [49], and attenuation of Fas-induced hepatocyte injury by anti-inflammatory agents underlines the role of inflammatory responses for DILI [50].

Because of the liver’s extraordinary capacities to regenerate, full recovery is even possible if severe DILI has already caused extensive hepatocyte death [51]. Different models of hepatotoxicity using a variety of hepatotoxins have demonstrated the crucial dynamic role of mitotic hepatocyte regeneration in response to toxic injury [52, 53]. The complex processes that promote hepatic tissue repair generally appear to show a dose-response relationship where stimulation of repair increases with higher exposure to hepatotoxins. However, above certain threshold toxicity also regenerative processes may be inhibited, and many factors modulate this critical switch from regeneration to unopposed injury, which determines the final outcome in DILI [54-56]. Mitochondrial GSH content appears to be such a modulating factor, underlining once more the key role of mitochondria in DILI [57, 58]. Although DILI can lead to chronic liver damage through special mechanisms such as chronic stimulation of fibrosis or targeted biliary toxicity with subsequent development of vanishing bile duct syndrome [59], the outcome of DILI is therefore mostly dichotomous, i.e. acute liver failure vs. full recovery.

#### Strengths and Limitations of the 3-Step Model

The 3-step model represents an attractive approach to hepatotoxicity for several reasons. An important feature is that it truly focuses on mechanisms and does not blend them with descriptive classifications, which eliminates some apparent contradictions that exist from other viewpoints. The model limits the principle mechanisms of DILI to three main ways of initial injury and on a vertical axis to three sequential main steps that lead to cell death. But at the same time it also integrates the crucial role of risk factors by appreciating the complex interactions of a large number of modulating factors and the fragile balance between injurious and protective factors that occur on all levels of the vertical axis (vide infra and Fig. (**2**)). In particular it emphasizes the importance of several amplification pathways and provides a plausible explanation for the rare idiosyncratic occurrence of severe DILI that develops on a background of frequent mild liver injury. In the absence of cofactors such mild injury may be necessary though not sufficient to cause severe DILI. But in the presence of cofactors mild injury can be amplified and then tip the balance of injurious vs. protective processes toward death pathways. This triggering role of cofactors is also able to plausibly explain the very long latency time that has been reported in some cases of DILI [60]. Furthermore, appreciation of the crucial role of downstream events determining the final outcome of DILI has two important implications for the definition of research targets in hepatotoxicity. First, agents that typically cause intrinsic DILI (e.g. APAP) can be used for the study of common downstream mechanisms that may also be relevant for idiosyncratic hepatotoxicity [61]. Second, downstream events represent potential targets for the development of new treatments that aim to interrupt pathways leading to apoptosis or necrosis.

At the same time any model is just a limited simplification of complex reality. Also the 3-step model should therefore be considered as a guiding working model that has limitations and may require adjustments to account for the ever-growing knowledge in mechanistic hepatotoxicity. Considering the complexity and interactions of the large number of often species-specific injurious vs. protective processes including metabolism, immune reactions and regeneration, a general concern is the extrapolation of data from animal or in-vitro models to humans. Demonstration of hepatotoxic mechanisms in such models does not necessarily imply that these also play a key role in human cases of DILI, particularly if concentrations of toxicants used in models do not reflect conditions in humans. Furthermore, although the model a priori suggests that its 3 vertical steps occur consecutively, one should be aware of the possibility that drugs may also directly affect downstream events. For example NAPQI has recently been shown to directly activate the Keap1-Nrf2 cell defense system [62-64]. Similarly, it may also be possible that drugs or their metabolites could directly stimulate other key elements in downstream pathways including death receptors.

## A MECHANISTIC VIEW ON RISK FACTORS

From a mechanistic point of view risk factors for hepatotoxicity are of particular interest, because they are the very explanation for the rare occurrence that defines idiosyncratic DILI. Severe idiosyncratic DILI typically occurs in less than 1 in 10’000 exposed patients, whereas most risk factors that have been associated with DILI have a much higher prevalence in the exposed population. In particular this is true for genetic polymorphisms, which, by definition, have a prevalence of at least 1%. Furthermore, even statistically significant relative risk estimates for such associations tend to be weak and therefore do not allow a reliable prediction of DILI in clinical practice. In perfect accordance with a complex multi-step mechanistic model of hepatotoxicity, this indicates that the presence of one single risk factor is usually not sufficient to explain the occurrence of idiosyncratic DILI, but that complex interactions of different mechanisms with several risk factors must be assumed in most cases. As mentioned above, incident environmental factors can function as triggering risk factors for DILI, particularly if they tip the balance of injurious vs. protective processes in the presence of other prevalent risk factors.

Several clinical risk factors for DILI such as high age, female gender and concomitant diseases or drugs have been described. Many of those may actually be associated with altered pharmacokinetics, which are an obvious risk factor whenever toxicity is dose-dependent and altered pharmacokinetics result in elevated concentrations of parent drugs and/or their toxic metabolites. However, from a current mechanistic point of view, it is most interesting to ask where risk factors exert their effect based on a multi-step model of hepatotoxicity that differentiates specific upstream vs. non-specific downstream mechanisms. In addition, risk factors can also be classified as either genetic or environmental. Such a view on risk factors superimposed on the 3-step model is shown in Fig. (**2**).

An intriguing implication of this view is that patients with risk factors that attenuate rather unspecific downstream events may have increased susceptibility to several hepatotoxins, and vice versa that common downstream risk factors could theoretically be identified in case-control studies that include idiosyncratic DILI cases caused by different hepatotoxins. Another interesting implication is that DILI could be caused by a different constellation of mechanisms and risk factors with different manifestations in different subjects. For example, a drug may be metabolized *via* CYP450 into a reactive metabolite with mild toxicity on mitochondria and also have some inhibitory effects on excretion of bile acids, resulting in a mild increase of liver enzymes in a given patient. Environmental factors such as a newly introduced concomitant drug or also ethanol may now induce CYP450. The toxicity of the metabolite then results in the death of a critical number of hepatocytes with a subsequent immune reaction against the reactive metabolite bound to hepatocyte proteins, and the patient develops severe DILI with symptoms and signs of an immune reaction. In another patient taking the same drug, a genetic variation of hepatic transporters plus preexisting impaired mitochondrial function associated with high age may be sufficient to lead to clinical manifestation of icteric liver disease.

Findings on idiosyncratic hepatotoxicity associated with herbal products containing kava (piper methysticum) may indeed be in accordance with this theoretical concept: several cases of acute liver failure during kava intake showed symptoms and signs of hypersensitivity indicating a specific immune reaction [65, 66]; at the same time there appears to be a background of more frequent increases in transaminases [67], and there is also evidence for a direct inhibition and uncoupling of mitochondrial respiratory chain and induction of apoptosis [68].

The complex role of different mechanisms and risk factors in hepatotoxicity can also be demonstrated by the example of APAP. APAP is a “classic” hepatotoxin for which an animal model is available, and also clinical APAP-induced DILI has been studied extensively. Toxification of APAP involves metabolism *via* CYP450 2E1, leading to its reactive metabolite NAPQI that causes oxidative cell stress unless it is detoxified by GSH. Once GSH stores are depleted covalent binding to proteins occurs. This was long considered as the principle direct and sufficient hepatotoxic mechanism. Several risk factors that relate to APAP metabolism and NAPQI-induced oxidative stress were consequently identified: ethanol can induce but also competitively inhibit the formation of NAPQI *via* CYP450 2E1 [10], ethanol or also starvation may contribute to critical GSH depletion [69, 70], inhibition of the constitutive androstane receptor (CAR) that regulates APAP metabolism including the formation of NAPQI can abrogate APAP-induced hepatotoxicity [71], and activation of NFE2L, a master regulator system for antioxidant defense that appears to be polymorphic in humans, protects against APAP toxicity [45, 72, 73]. However, recent research focused on further downstream events, and subsequently demonstrated that these also play a key role in APAP hepatotoxicity. Several studies showed that JNK, TNFα, IFNγ, FasL, IL10 and IL6 have a major impact on the final outcome following the formation of NAPQI and consumption of GSH [31-33, 74-78]. Furthermore, several studies suggest that GSH also plays an important role in the regulation of downstream events: GSH depletion appears to increase injury in models of TNFα-induced liver cell injury [47, 70, 79]. The mechanisms are not entirely clear but may involve increased activation of stress kinases, decreased defense against mitochondrial oxidative stress, or inhibition of TNFα-induced NF-κB activation [80]. Furthermore, ethanol may also have a role in APAP-induced DILI through an impairment of mitochondrial function [81], and after a potential role of transport proteins in DILI was realized, also hepatobiliary efflux transporters were shown to affect APAP-induced hepatotoxicity [82].

Studies of APAP-induced hepatotoxicity clearly demonstrate the role of many environmental risk factors and the role of toxic metabolites, oxidative stress and defense against it with a crucial role for GSH, and the contribution of both, initial toxic cell stress, as well as subsequent downstream events, for DILI. The relevance of other risk factors, particularly genetic factors, has been shown with hepatotoxins that typically cause idiosyncratic hepatotoxicity. Like environmental factors, genetic variations can principally affect all mechanisms involved in DILI, from drug metabolism over initial injury and mitochondrial impairment, to cell death and regeneration.

Examples of genetic factors that affect drug metabolism or detoxification and have been associated with an increased risk of DILI are genetic variations in the GSH S-transferases GSTT1 and GSTM1, CYP450 2E1, N-acetyltransferase, as well as manganese superoxide dismutase (SOD2) [83-86]. Interestingly SOD2 resides in mitochondria, and troglitazone toxicity has been investigated in a SOD2+/- mouse model [87]. DILI occurring with hypersensitivity has also been associated with genetic polymorphisms that involve drug toxification or detoxification, which also suggests that dose plays a role in immune-mediated DILI [88, 89]. This is further supported by a correlation between quantitative differences in the metabolic toxification of different inhalational anesthetics and their associated risk for development of autoimmune hepatotoxicity [90]. HLA genotype variations have also been associated with allergic hepatotoxicity [91], although their predictive value does not appear to be as high as for HLA variations linked to abacavir-induced hypersensitivity reactions [92, 93].

Cytokine polymorphisms associated with diclofenac-induced hepatotoxicity are another genetic risk factor of particular interest, because they relate to a downstream mechanism in DILI and could therefore also be relevant for other idiosyncratic hepatotoxins [94]. The same would apply to genetic variations involving mitochondrial function, and interestingly genetic deficiency of mitochondrial long-chain 3-hydroxyacyl-CoA dehydrogenase has been associated with acute fatty liver of pregnancy, which is presumably related to increased levels of female sex hormones [95, 96]. However the relevance of this very rare variation for hepatotoxicity associated with estrogens or other drugs that inhibit mitochondrial β-oxidation such as amiodarone is unclear. However, genetic mitochondrial abnormalities with subsequently increased susceptibility to hepatotoxins may also be age-related and therefore be a possible explanation of increased risk of DILI with higher age [97-99]. Another environmental risk factor for DILI that acts *via* an impairment of mitochondrial function could be obesity and diabetes associated with steatosis and steatohepatitis [100-103].

Other genetic risk factors that recently gained much attention are determinants of hepatic transport proteins. Genetic (or environmental) factors that affect basolateral (sinusoidal) or apical (canalicular) transport systems will determine exposure of hepatocytes not only to toxic drugs but also to toxic bile constituents and therefore represent both, risk factors as well as mechanisms for cholestatic DILI [17, 104-108]. Involvement of hepatic transporters has been reported in DILI induced by troglitazone, hormonal contraceptives, or bosentan [106, 108-110]. For example, the V444A polymorphism in the BSEP (*ABCB11*) gene has been associated with an increased risk of DILI [107, 111], which may even affect drugs that do not typically cause a cholestatic pattern of injury. Furthermore, recently also variations in nuclear receptors PXR and CAR which not only regulate hepatic drug metabolism and disposition but also recognize bile acids have been identified and these may also modulate the risk for DILI with or without involvement of bile-acid induced injury [112].

Considering the important role of inflammatory mediators that can sensitize hepatocytes to injurious effects to cytokines (vide supra), it is not surprising that inflammation has not only been shown to enhance hepatotoxicity in an animal model [113], but also that a number of concomitant infectious diseases have been identified as clinical risk factors for DILI. Aspirin-induced liver failure in children with an inhibition of mitochondrial β-oxidation in the presence of infection (Reye syndrome) is a well-known example [114, 115]. But also patients undergoing antiretroviral treatment for HIV infection have a higher risk of severe hepatotoxicity when co-infected with the hepatitis B or C viruses, particularly if the regimen contains protease inhibitors [116, 117]

## FUTURE PERSPECTIVES

A better understanding of mechanisms of hepatotoxicity is a major future challenge in order to develop new solutions for the prevention and treatment of DILI. Recent important advances in the understanding of general hepatotoxic mechanisms are the foundation for further studies on mechanisms for specific hepatotoxic drugs. The increased appreciation of the crucial role and complex regulation of downstream events that modulate the critical switch from recovery to unopposed injury also implies that they represent promising new therapeutic targets. For example, specific JNK inhibitors are in clinical development for the treatment of APAP-induced hepatotoxicity, and they could also be protective against other hepatotoxins. Other potential downstream targets are the inhibition of any amplifying pathways, e.g. through an inhibition of injurious or a stimulation of protective cytokines and Bcl-2 proteins. Also specific caspase inhibitors that may prevent apoptosis are a possible approach. Given that apoptosis activates stellate cells leading to liver fibrosis, attenuation of apoptosis may even lead to the resolution of fibrosis. In fact, according to some reports, the beneficial effects of ursodeoxycholic acid are partly mediated through an inhibition of apoptosis by modulating MPT [118, 119]. Nevertheless, one should be aware that apoptosis is not necessarily an unwanted mode of cell death as it is associated with little collateral damage. A new therapeutic approach that aims to inhibit apoptosis may therefore theoretically also result in a shift towards necrosis with additional collateral damage. Also the recently gained knowledge that GSH has a protective role not only *via* its action on upstream events, such as the prevention of NAPQI-induced initial injury, but also *via* its effect on downstream processes that are not specific to APAP toxicity may justify trials that evaluate possible beneficial effects in the treatment of other forms of DILI including idiosyncratic hepatotoxicity.

Knowledge of genetic and environmental contributors that determine actual idiosyncratic DILI in humans is necessary in order to focus mechanistic in-vitro and animal toxicology studies. Likewise, also prevention of DILI in clinical practice is inevitably linked to the identification of risk factors that predict severe DILI with sufficient sensitivity and specificity. Pharmacogenetic approaches such as case-control studies that consider interactions between genetic but also environmental factors may be able to reach that goal. However, the complexity of interactions being sufficient to cause severe DILI, the difficulties in the reliable identification of environmental risk factors, and also in the reliable causality assessment of clinical cases of DILI must not be underestimated. Therefore it is currently unknown whether this is a realistic scenario for clinically important hepatotoxins.

Considering the importance of hepatotoxic metabolites, strategies aimed at identifying early markers of DILI that may precede ALT elevations are focusing on the use of metabonomics, e.g. high resolution NMR of urine samples. Patients that go to develop elevated ALT levels may have a different metabolome, and the spectra obtained before ALT values exceed the upper limit of normal might indicate a predictive metabolome signature. In fact, trials conducted with known idiosyncratic hepatotoxins such as isoniazid could be used to identify “Hy’s law” cases in serial collections [120, 121], allowing for metabonomics in well characterized cases.

Identification of risk factors for rare idiosyncratic hepatotoxicity requires special networks that contribute to data collection and subsequent identification of environmental as well as genetic risk factors for clinical cases of idiosyncratic DILI. With that in mind the DILI network project links several university medical centers that collect detailed information on cases of DILI including standardized causality assessments and samples for genetic analyses. The data collection focuses on idiosyncratic hepatotoxicity and particularly aims to identify upstream as well as downstream genetic determinants of hepatotoxicity [122, 123]. Furthermore, the recognition that even very rare idiosyncratic DILI typically occurs on a background of more frequent mild increases in transaminases makes it possible to identify necessary (although not sufficient) risk factors in controlled cohorts from prospective clinical trials. Genome wide association studies that analyze several hundred thousand single nucleotide polymorphisms (SNPs) per patient have now become technically and economically feasible and have been successfully used for the identification of genetic risk factors even of rare adverse drug reactions in large phase III clinical trials [124]. Given the economical and regulatory pressure associated with severe idiosyncratic hepatotoxicity that is only identified after marketing of a new drug, such pharmacogenomic approaches will likely also be used for the identification of risk factors associated with DILI during clinical drug development.

From a clinical standpoint the important role of triggers and risk factors that may be required in idiosyncratic hepatotoxicity should stimulate clinicians to search for such factors when collecting, analyzing and reporting data of individual cases and case series of DILI. Furthermore, the increasing recognition of the hepatotoxic potential of herbal products should alert physicians as well as consumers. In fact it is well possible that a substantial proportion of cases of so-called “idiopathic” acute liver failure are indeed due to herbal hepatotoxicity. Every clinician should therefore include herbal products of any kind in the differential diagnosis and actively search for them when taking the history of a patient with liver disease of unclear origin. From a regulatory point of view the issue of herbal hepatotoxicity also raises the question whether not only herbal drugs but also herbal dietary supplements may generally require a stricter regulation of their distribution and use [4-6]. For diagnostic purposes it is also of interest that the recognition of apoptosis as an important mode of cell death in DILI implies that new markers for apoptosis such as the monoclonal M30 antibody, which recognizes a caspase-generated cytokeratin 18 neoantigen, may be useful in the early diagnosis of DILI [125, 126]. Nevertheless, DILI currently remains unpredictable for most hepatotoxins, and given that APAP is the only hepatotoxin for which there is a proven therapy for the prevention of DILI available, every clinician should be reminded to avoid unnecessary drug use and to stop suspicious drugs immediately as soon as symptoms and signs of DILI are identified.

## Figures and Tables

**Fig. (1):**
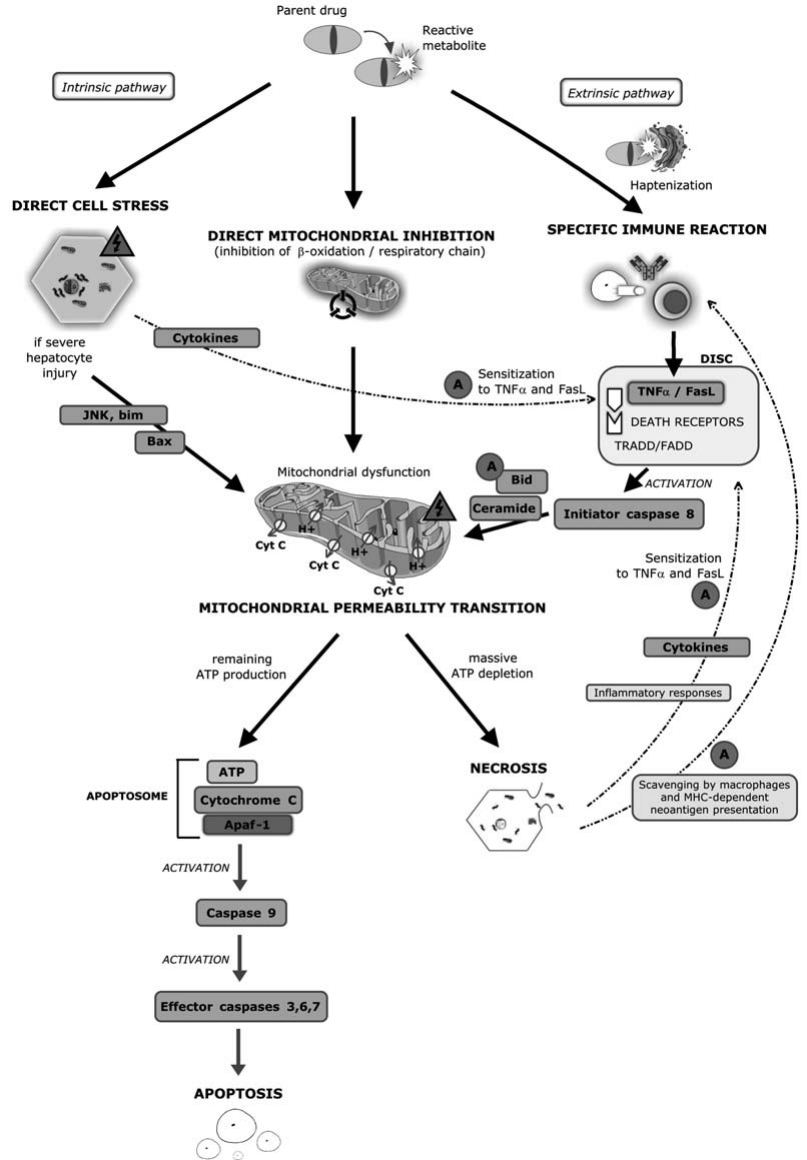
A 3-step mechanistic working model of hepatotoxicity. First, initial injury is exerted through direct cell stress, direct mitochondrial inhibition and/or specific immune reactions. Second, initial injury can lead to mitochondrial permeability transition (MPT). Direct cell stress causes MPT *via* the intrinsic pathway. The intrinsic pathway involves activation of intracellular stressor cascades and pro-apoptotic proteins including Bax. Alternatively MPT can be initiated through the death receptor-mediated extrinsic pathway that is activated by immune reactions and/or after sensitization to TNFα and FasL binding to death receptors. Cytokines modulate the sensitivity of its activation. Third, MPT leads to necrosis or apoptosis depending on the availability of ATP. In hepatocytes activation of initiator caspase 8 through the extrinsic pathway is not sufficient to directly activate apoptosis, but amplification through pro-apoptotic factors including Bid and ceramides lead to MPT, which will then lead to the apoptotic pathway that is activated in the presence of sufficient remaining ATP production. Necrosis occurs if there is no ATP available, which is required for energy-consuming apoptotic pathways. Several highlighted amplification mechanisms (A) may play an important role at different levels for the idiosyncratic occurrence of hepatotoxicity.

**Fig. (2):**
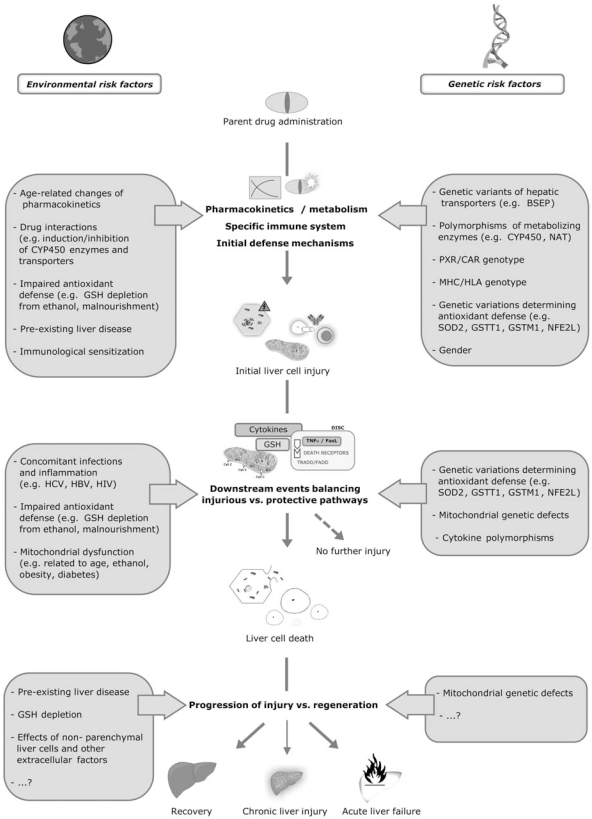
Risk factors for hepatotoxicity. First, risk factors can be classified into environmental vs. genetic factors. From a mechanistic point of view risk factors can further affect all different levels of events leading to the final outcome of drug-induced liver injury, which is mostly dichotomous, i.e. full recovery vs. acute liver failure. Note that risk factors affecting events downstream of initial injury are rather unspecific regarding different hepatotoxins. The figure presents a selection of well-described risk factors, but one must assume that other factors also play a role, of which many currently remain unknown.

## ABBREVIATIONS

APAP: = Acetaminophen
ATP: = Adenosine triphosphate
BSEP: = Bile salt efflux pump
CYP450: = Cytochrome P450
DILI: = Drug-induced liver injury
DISC: = Death-inducing signaling complex
GSH: = Glutathione
GST: = Glutathione S-transferase
IF: = Interferon
IL: = Interleukin
JNK: = C-jun N-terminal kinase
MHC: = Major histocompatibility complex
MPT: = Mitochondrial permeability transition (opening of the “MPT pore” in the inner mitochondrial membrane)
NAPQI: = N-acetyl-p-benzoquinone imine
NF-κB: = Nuclear transcription factor kappa B
NFE2L (Nrf2): = Nuclear factor erythroid-derived 2-like
ROS: = Reactive oxygen species
SOD2: = Manganese superoxide dismutase
TNFα: = Tumor necrosis factor alpha
